# When and How to Regulate: Everyday Emotion-Regulation Strategy Use and Stressor Intensity

**DOI:** 10.1007/s42761-021-00087-1

**Published:** 2021-12-10

**Authors:** Elisabeth S. Blanke, Jennifer A. Bellingtier, Michaela Riediger, Annette Brose

**Affiliations:** 1 Department of Psychology, Humboldt-Universität Zu Berlin, 10099 Berlin, Germany; 2 Department of Developmental Psychology, Friedrich-Schiller-Universität Jena, Jena, Germany; 3 German Institute for Economic Research (DIW Berlin), Berlin, Germany

**Keywords:** Emotion regulation choice, Emotion regulation flexibility, Experience sampling, Stressor intensity, Situation-strategy fit (5/6)

## Abstract

Contextual factors shape emotion regulation (ER). The intensity of emotional stimuli may be such a contextual factor that influences the selection and moderates the effectiveness of ER strategies in reducing negative affect (NA). Prior research has shown that, on average, when emotional stimuli were more intense, distraction was selected over reappraisal (and vice versa). This pattern was previously shown to be adaptive as the preferred strategies were more efficient in the respective contexts. Here, we investigated whether stressor intensity predicted strategy use and effectiveness in similar ways in daily life. We examined five ER strategies (reappraisal, reflection, acceptance, distraction, and rumination) in relation to the intensity of everyday stressors, using two waves of experience-sampling data (*N* = 156). In accordance with our hypotheses, reappraisal, reflection, and acceptance were used less, and rumination was used more, when stressors were more intense. Moreover, results suggested that distraction was more effective, and rumination more detrimental the higher the stressor intensity. Against our hypotheses, distraction did not covary with stressor intensity, and there was no evidence that reappraisal, reflection, and acceptance were more effective at lower levels of stressor intensity. Instead, when examined individually, reflection and reappraisal (like distraction) were more effective at higher levels of stressor intensity. In sum, stressor intensity predicted ER selection and moderated strategy effectiveness, but the results also point to a more complex ER strategy use in daily life than in the laboratory.

**Supplementary Information:**

The online version contains supplementary material available at 10.1007/s42761-021-00087-1.

Emotion regulation (ER) describes various processes pertaining to the modulation of emotions (Gross, 1998). According to recent theory, a cornerstone of adaptive ER is the ability to flexibly adjust ER strategies to a given context, in line with one’s goals (Aldao et al., 2015; Bonanno & Burton, 2013). The ER choice framework (Sheppes, 2020) introduced intensity of emotional information (e.g., the intensity of emotional stimuli such as a sad pictures or movies) as one contextual feature that influences ER strategy selection and effectiveness. Here, ER effectiveness refers to reducing negative affect (NA), which is one of the most prominent goals of ER use (Riediger et al., 2009).

Specifically, the ER choice framework proposes that individuals should be more likely to select distraction when presented with highly emotionally intense stimuli over reappraisal (Sheppes, 2020). This may be the case, because when emotional information is highly intense, individuals experience higher arousal, and their attentional and cognitive resources may be focused on the event, likely making it difficult to perform other cognitively demanding tasks (Veilleux et al., 2021). According to the process model of ER, reappraisal entails engaging with the emotional content and changing its meaning (Gross, 1998, 2015). As this requires cognitive resource expenditure, reappraisal should not only be selected less when dealing with highly emotionally intense information, but it should also be less likely to be effective (as compared to situations with low intensity information). This means that emotional intensity may not only guide the selection, but also moderate the effectiveness of ER strategies (Sheppes, 2020). In contrast, and also proposed by the ER process model, attentional disengagement strategies, such as distraction, are aimed at diverting attention from emotional information. This may require less cognitive resource expenditure, so that individuals select distraction more readily in the face of emotionally intense information, and it may also be more effective in regulating NA than reappraisal in this situation (Sheppes, 2020).

There is some empirical evidence for these propositions. For example, participants in a lab study described reappraisal as more effortful to implement than acceptance (Troy et al., 2018). Sheppes and Meiran (2008) found that participants performed worse in a Stroop task after using reappraisal than using distraction, suggesting exhaustion as a mechanism. Furthermore, people who used distraction (compared to reappraisal) had more problems remembering target stimuli in this study, suggesting that distraction is associated with less deep processing than reappraisal. Overall, these results may help to explain why participants tend to select distraction over reappraisal (and vice versa) when emotional stimuli were more intense (e.g., Sheppes et al., 2014). Moreover, selecting strategies in such a way was shown to be effective in reducing NA (e.g., Sheppes & Meiran, 2007; for an overview, see Sheppes, 2020).

While research under standardized conditions is of great importance, it is pivotal to also investigate ER in everyday life, in which individuals face personally meaningful situations and are not instructed to use certain strategies. In this study, we investigated the intensity of stressors (i.e., subjective appraisals of the severity of negative events) as an indicator of the intensity of emotional information in everyday life. In line with the ER choice paradigm, we expected that ER strategy use and effectiveness would vary depending on stressor intensity.

Previous research in daily life, using the experience-sampling method (ESM) or daily diaries, has provided mixed evidence for the notion that ER-strategy use covaries with stressor-related NA. In line with propositions of the ER choice framework, a preferred use of distraction to regulate higher intensity stressor-related NA in everyday life was found in three studies (in adolescents, Lennarz et al., 2019; and in adults, Mehta et al., 2020; Troy et al., 2019). Reappraisal was used more when affective intensity was lower in two of these studies (Mehta et al., 2020; Troy et al., 2019) and in one additional study (Wilms et al., 2020). Furthermore, participants reported more exhaustion after using reappraisal than after using mindfulness (including acceptance) in an ESM study (Wenzel et al., 2021a).

However, other studies have provided conflicting evidence. Stressor-related intensity of NA was unrelated to distraction in one study (Wilms et al., 2020). In a study subsuming strategies to categories, stressor-related NA intensity was associated with *reduced* use of strategies including distraction and unrelated to strategies including reappraisal (Ortner & Pennekamp, 2020). Furthermore, reappraisal was unrelated to stressor-related NA intensity in a study with adolescents (Lennarz et al., 2019).

Overall, these studies differed in many ways from one another, such as in how strategies were defined (e.g., single strategies vs. subsuming strategies), how many and which strategies were researched (e.g., three to twenty-two), what kind of stressors were researched (e.g., recurring stressors vs. everyday stressors), as well as studies’ sampling frequency (daily vs. every other hour). Furthermore, these previous studies have usually inferred stressor intensity from the intensity of stressor-related NA. However, this conflates stressor intensity with the emotional reaction to the stressor, making it difficult to examine strategies’ effectiveness. In sum, additional research is needed to further elucidate the role that intensity variations may have for strategy selection and effectiveness in daily life.

Given this background, the first aim of the present study was to investigate associations between stressor intensity and the selection of various ER strategies in daily life, using data from two waves of a longitudinal ESM study. In accordance with the ER choice framework (Sheppes, 2020), we investigated distraction and reappraisal. Following Sheppes (2020), we categorized distraction as an attentional disengagement strategy, which should be selected *more* when stressors are more intense (see Table 1 for an overview). The opposite should be the case for reappraisal, an engagement meaning-change strategy (Sheppes, 2020), which should be selected *less* when stressors are more intense.

**Table 1:** Overview of ER strategies, underlying assumptions, and hypotheses

Strategies	Underlying assumptions		Hypotheses	
	Strategy categorization	Cognitive resource expenditure	Stressor intensity when strategy is likely to be selected	Stressor intensity when strategy is likely to be effective in reducing NA
Established strategies in the ER Choice paradigm				
Distraction	Attentional disengagement	Low	High	High
Positive reappraisal	Engagement meaning-change	High	Low	Low
Additional strategies				
Acceptance	Attentional engagement	Medium	Low	Low
Reflection	Attentional engagement	Medium	Low	Low
Rumination	Attentional engagement	Medium	High	(None)

Additionally, we investigated three other cognitive^1^ ER strategies commonly examined in ESM studies (e.g., Brans et al., 2013), namely, acceptance, reflection, and rumination. Acceptance has been included in previous research on ER choice, but, to our knowledge, reflection and rumination have not. Whereas reflection refers to putatively adaptive thought patterns about an emotional event, rumination refers to putatively maladaptive thought patterns (e.g., Blanke, et al., 2020c). Similar to reappraisal, these three strategies can be categorized as engagement strategies (i.e., strategies that are aimed at concentrating on the emotional event), but unlike reappraisal, they are not meaning-change strategies. Instead, these strategies can be viewed as attentional engagement strategies (e.g., Blanke, et al., 2020c; Troy et al., 2018). Thus, they may fall in between reappraisal and distraction in terms of cognitive resource expenditure.

The consideration of these additional strategies provides potentially interesting insights. By including other engagement strategies, we can test whether people truly want to disengage (and thus chose distraction over other engagement strategies) or whether they are just not able to reappraise, but potentially use other less difficult strategies (such as reflection or acceptance). Possibly, meaning-change may be too difficult to implement in intense situations, but other engagement strategies may not be. Alternatively, people may resort to rumination when they want to engage, even though rumination may not be effective.

Previous research on ER choice in other strategies than distraction and reappraisal is rare, and results are mixed. Whereas the selection of acceptance was not predicted by stressor intensity in a lab study (Mehta et al., 2017), it was more strongly endorsed when stressor-related NA was lower in two studies in daily life (Lennarz et al., 2019; Mehta et al., 2020). For reflection, we are not aware of previous findings regarding its selection in relation to stressor-related NA intensity. We tentatively expected a similar pattern for acceptance and reflection as for reappraisal—less use when stressor intensity increases—as these engagement strategies may be cognitively costlier than attentional disengagement strategies, such as distraction. Unlike the other strategies, rumination can be rather uncontrollable (Raes et al., 2008) and may be associated with lower cognitive control (Beckwé et al., 2014). Rumination was already shown to be associated with higher stressor-related NA (e.g., Ortner & Pennekamp, 2020), and it is the only one of the discussed strategies for which there is direct evidence that it is endorsed more when stressors are more intense in daily life (e.g., Genet & Siemer, 2012; Vanderhasselt et al., 2016). For example, a study with customer service employees showed that the intensity of their rumination depends on the frequency of stressful customer maltreatment (Wang et al., 2013). We therefore expected to replicate a positive association between rumination and stressor intensity. Taken together, we hypothesized that acceptance, reflection, and reappraisal would be used less, whereas distraction and rumination would be used more the higher the stressor intensity (H1).

A second aim of this study was to address whether the intensity of stressors moderates the effectiveness of ER strategy use in daily life—a question that previous ESM studies have not yet addressed, to the best of our knowledge. In general, a meta-analysis across studies in the lab found that reappraisal, acceptance (here conceptualized as a form of reappraisal), and forms of distraction are effective in reducing NA in general, whereas reflective strategies (including rumination) were associated with increases in NA (Webb et al., 2012). In daily life, reappraisal, acceptance, and sometimes distraction were repeatedly shown to be associated with reduced NA in the face of stressors (Lennarz et al., 2019; Mehta et al., 2020; Troy et al., 2019). Reflection and reappraisal were also shown to be associated with increased positive affect in daily life more broadly (Brans et al., 2013). Rumination, instead, was associated with increased NA (e.g., Genet & Siemer, 2012).

In the present study, we hypothesized that stressor intensity would moderate ER in the prediction of stressor-related NA^2^: We hypothesized that the effectiveness of distraction (i.e., its negative association with NA) and the detrimental effect of rumination (i.e., its positive association with NA) would be particularly pronounced when dealing with higher (vs. lower) intensity stressors; all other strategies should be more effective (negatively associated with NA) when dealing with lower (vs. higher) intensity stressors (H2).

Table 1 summarizes the hypotheses. Reappraisal, acceptance, and reflection were thought to be both selected and effective in situations low in stress intensity, as these strategies are engagement strategies that require some cognitive resource expenditure. Distraction was thought to be selected and effective in high-stress situations. Thus, we expected selection and effectiveness to converge for these strategies. Rumination was thought to diverge from this pattern, as it has been shown to be a strategy that is selected in highly stressful situations without being effective. This points toward individuals either not being aware that rumination is ineffective or not being in control when using this strategy.

Beyond extending evidence on the ER choice framework by using an ESM approach, our study contributes to previous research in the following ways: We used data from two waves of experience sampling, thus investigating many and various stressor occasions. This should make our results particularly robust. Furthermore, we investigated five strategies and directly measured stressor intensity instead of inferring it from emotional reactions. Lastly, we investigated whether stressor intensity moderated strategy effectiveness (in line with assumptions of the ER choice paradigm).

## Method

### Participants and Procedures

We used data from the EE-SOEP-IS study (Everyday Experiences [EE] in the Innovation Sample [IS] of the Socio-Economic Panel Study [SOEP]; Richter, & Schupp, 2015; Siebert et al., 2017). The EE-SOEP-IS study consisted of two waves of ESM, approximately 1 year apart (for details, see Siebert et al., 2017). The principal investigator of the study (A.B.) aimed for a sample size of *N* = 180 for Wave 1, which was almost achieved (*N* = 179). This sample size was based on previous experiences with ESM research, and in accordance with the primary hypotheses of the initial research proposal, which are not part of the present paper. Other findings from this dataset are published in Blanke, Brose, et al (2020); Blanke, Kalokerinos, et al (2020); Blanke, Schmidt, et al (2020) and Wenzel, Blanke, Rowland & Brose (2021); Wenzel, Blanke, Rowland, & Kubiak (2021). In both waves, middle-aged participants were visited at their homes by interviewers from the Humboldt-Universität zu Berlin. At the home sessions, participants worked on questionnaires and tasks (which are of no relevance for the present study) presented on laptops. Interviewers provided the participants with smartphones (Huawei Ascend G330) programmed with a custom-made ESM program (see also Riediger et al., 2009). The day after the home sessions, the ESM phases started, which spanned 3 weeks each, consisting of three blocks of four sampling days. Participants chose a 12-h time-frame for the sampling days (e.g., from 8 a.m. to 8 p.m.). At each sampling day, participants semi-randomly received six ESM prompts (beeps). The 4-day assessment blocks were prolonged by up to 2 days if participants completed less than five beeps a day. Participants received 170 to 190 Euros for participation in the two waves, depending on their participation in the ESM.

Of the 179 participants who participated in Wave 1, 156 participants provided ESM data for both waves (53% women) and were included in the analyses. They were aged between 38 and 61 years (*M* = 50.74, *SD* = 5.85) at Wave 1. The 23 participants not included in the analyses did not differ from the continuing sample in terms of age, gender composition, experience of NA, or stressor intensity. Mann–Whitney *U* tests revealed little evidence for differences in average strategy use (*p* > .05 for all strategies except for distraction, *p* = .046; participants who continued the study endorsed distraction more).

In the present study, we only focused on measurement occasions during which participants reported that a stressor had occurred since the last beep/since waking up.^3^ In Wave 1, participants reported 20.5 stressor occasions on average (*SD* = 15.9; range 1–68); in Wave 2, they reported 20.3 stressor occasions on average (*SD* = 17.9; range 0–70; five individuals did not report any stressors).^4^ The study and the analysis plan were not preregistered.

### ESM Measures

ESM measures were identical for both waves. Descriptive information for each wave including individuals’ means, standard deviations, and the intra-class correlations (ICCs) at stressor occasions are reported in Table 2. To summarize associations, correlations between variables across both waves (at the level of the individual) are reported in Table 3. We reported Spearman’s correlations to accommodate for deviations from the normal distribution.

**Table 2:** Descriptive statistics for Both ESM waves based on occasions when a stressor occurred

Variables	Wave 1		Wave 2	
	*iM* (*SD*)	ICC	i*M (SD)*	ICC
Negative affect	1.48 (0.93)	.43	1.43 (0.95)	.48
Stressor intensity	3.84 (1.04)	.30	3.83 (0.99)	.34
Rumination	2.38 (1.16)	.33	2.31 (1.11)	.29
Distraction	2.41 (1.20)	.38	2.46 (1.30)	.44
Acceptance	3.18 (1.17)	.36	3.28 (1.19)	.37
Reflection	3.00 (1.14)	.38	3.00 (1.27)	.43
Reappraisal	2.41 (1.31)	.42	2.26 (1.35)	.44

*n* = 5 individuals did not report any stressors in Wave 2

*iM* individual mean; *SD* standard deviation; ICC intraclass correlation

**Table 3:** Spearman’s correlations of the study variables at the level of the individual (across waves)

Variables	1	2	3	4	5	6	7
1. Negative affect		.26	.59	.22	-.20	-.16	.12
2. Stressor intensity	.27		.40	.12	.10	.01	.01
3. Rumination	.37	.36		.28	-.17	-.13	.04
4. Distraction	-.03	.00	.07		.23	.41	.59
5. Acceptance	-.07	-.05	-.08	.19		.42	.18
6. Reflection	-.15	-.14	-.09	.20	.20		.65
7. Positive reappraisal	-.07	-.12	-.05	.24	.07	.40	

Above the diagonal: between-person correlations; below the diagonal: average within-person correlations

### Stressor Occurrence and Intensity

At each beep, participants were asked whether a stressor had occurred since the last beep or since waking up (at the first beep of the day). Participants either answered *yes* (coded 1) or *no* (coded 0). Only stressor occasions are included in these analyses. When participants reported a stressor, they were asked to rate on a 7-point scale how much this stressor affected them when it occurred, ranging from 0 — *barely* to 6 — *very much*, which serves as an indicator of stressor intensity in this study.

#### NA

Negative affect (NA) was assessed asking participants “How are you feeling right now?” Six NA items were selected to reflect different levels of arousal (including PANAS items, Watson et al., 1988) and were rated on a 7-point scale ranging from 0 — does not apply at all to 6 — applies strongly. The items were nervous, jittery, angry, upset, downhearted, and distressed. Within-person reliability estimates (McDonald’s Omega; Geldhof et al., 2014) for the six items at stressor occasions were .71 at Wave 1 and .70 at Wave 2.^5^

#### ER

Participants were asked to “Think about the most unpleasant or stressful things/feelings you have had since the last beep (at the first beep of the day: since you woke up). How did you handle them?” Then, participants rated five emotion regulation (ER) strategies on a 7-point scale from 0 — does not apply at all to 6 — applies strongly. The strategies were rumination (“I could not stop thinking about it”), distraction (“I distracted myself from the distressing things and feelings”), acceptance (“I accepted the things /feelings”), positive reappraisal (“I searched for positive aspects of this matter”), and reflection (“I thought about it in a calm and relaxed fashion”).^6^

### Data Analysis

Data was prepared and analyzed in IBM SPSS Version 25 for Windows, Mplus Version 8.3, and SAS Version 9.4. For our analyses, we used multilevel modeling (MLM) with restricted maximum likelihood (REML) estimation. The models were three-level models with beeps (level 1) nested within waves (level 2), and waves nested within individuals (level 3). We did not have any hypotheses regarding wave-level differences, and thus simply controlled for the fact that these were different bursts. For interested readers, we also report the results for each wave when analyzed separately in the supplementary materials (Tables S1-S3). In all analyses, we controlled for potential time-related trends by entering a variable that indicated the number of days passed since their first scheduled beep (starting at 0). All other predictor variables were centered at individuals’ means within each wave to be able to investigate within-person and within-wave effects. When testing H1, we accounted for the autoregressive, unequally spaced time spans between measurement occasions by using the spatial power error structure function in SAS. When testing H2, we investigated change in NA by entering lagged NA (NA at the previous measurement occasion) as a predictor (thus modeling autoregression directly without specifying an autoregressive error structure). Random intercepts were included at both levels (levels 2 and 3). As including random slopes (and covariations of other random effects) for all variables in complex models is often not possible (due to convergence issues), we included random slopes (and their covariation with the intercept and other slopes) at both levels for the time trend and stressor intensity when possible. In cases in which lagged affect was included, this was also modeled including a random slope when possible.

## Results

### Strategy Selection and Stressor Intensity (H1)

To investigate strategy selection (H1), ER strategies were treated as outcomes and stressor intensity as the predictor (in SAS). We expected individuals’ strategy use in daily life to be associated with stressor intensity. In accordance with our hypothesis, more intense stressors were associated with a stronger endorsement of rumination, and a weaker endorsement of acceptance, reflection, and reappraisal (Table 4). Thus, when stressors were more intense, individuals ruminated more, and accepted, reflected, and reappraised less. Contrary to our hypothesis, distraction was not endorsed more when stressors were more intense. That is, distraction was used regardless of stressor intensity.

**Table 4:** Selected fixed effects from 3-level models: associations between strategy selection and stressor intensity (separate analyses per strategy)

Dependent variables	Estimate	95% CI		*p*
		*LL*	*UL*	
Rumination	**0.449**	0.396	0.502	** < .001**
Distraction	− 0.017	− 0.060	0.027	.447
Acceptance	− **0.091**	− 0.141	− 0.040	** < .001**
Reflection	− **0.167**	− 0.216	− 0.117	** < .001**
Reappraisal	− **0.164**	− 0.206	− 0.122	** < .001**

Random slopes were estimated at both levels (wave level and person level) for elapsed days and stressor intensity. In the model for reappraisal, the slope for elapsed days was not estimated as a random slope at level 3 due to convergence issues. Bold print indicates significant effects (*p* < .05) as relevant for the hypotheses

*CI* confidence interval, *LL* lower limit, *UL* upper limit

As strategies are interrelated (see Table 3), we set up an alternative multilevel path model (in M*plus*, using the standard maximum likelihood with robust standard errors (MLR) estimator), letting the residuals of all strategies covary. The pattern of results remained the same, and the estimates were similar (see Table S1b).

### Strategy Effectiveness and Stressor Intensity (H2)

To investigate whether ER strategies’ effectiveness was moderated by stressor intensity, we examined within-person interactions. Specifically, we examined whether the effects of strategies on the change in NA (i.e., by controlling for lagged NA) interacted with stressor intensity. In a first set of analyses, we examined the effect of all strategies separately to investigate the individual role of each strategy (Table 5). As strategies may share variance, we then ran a combined model, meaning that we tested for the effect of the strategies above and beyond all others (Table 6).

**Table 5:** Selected fixed effects from 3-level models (separate models per strategy): associations between NA controlling for lagged NA (change in NA), strategies, and stressor intensity

	Estimate	95% CI		*p*
Models		*LL*	*UL*	
Rumination model				
Stressor intensity	0.148	0.118	0.178	< .001
Rumination	0.174	0.158	0.190	< .001
Stressor intensity × rumination	**0.044**	0.033	0.056	** < .001**
Distraction model				
Stressor intensity	0.216	0.185	0.247	< .001
Distraction	− 0.051	− 0.068	− 0.034	< .001
Stressor intensity × distraction	** − 0.023**	− 0.036	− 0.010	** < .001**
Acceptance model				
Stressor intensity	0.212	0.181	0.243	< .001
Acceptance	− 0.059	− 0.076	− 0.042	< .001
Stressor intensity × acceptance	− 0.008	− 0.020	0.005	.241
Reflection model				
Stressor intensity	0.205	0.173	0.236	< .001
Reflection	− 0.085	− 0.102	− 0.067	< .001
Stressor intensity × reflection	** − 0.014**	− 0.027	− 0.001	**.033**
Reappraisal model				
Stressor intensity	0.210	0.178	0.241	< .001
Reappraisal	** − **0.043	** − **0.060	** − **0.027	< .001
Stressor intensity × reappraisal	** − 0.015**	** − **0.027	** − **0.002	**.022**

Random effects were estimated at both levels for elapsed days, lagged NA, and stressor intensity. Bold print indicates significant effects (*p* < .05) relevant for the hypotheses

*NA* negative affect, *CI* confidence interval, *LL* lower limit, *UL* upper limit

**Table 6:** Fixed effects from 3-level model (combined model): associations between NA controlling for lagged NA (change in NA), strategies, and stressor intensity

	Estimate	95% CI		*p*
Parameters		*LL*	*UL*	
Intercept	1.489	1.352	1.627	< .001
Days in study	− 0.008	− 0.013	− 0.003	< .001
Lagged NA (*t* − 1)	0.195	0.156	0.234	< .001
Stressor intensity	0.134	0.105	0.163	< .001
Rumination	0.168	0.151	0.184	< .001
Distraction	− 0.031	− 0.048	− 0.015	< .001
Acceptance	− 0.020	− 0.036	− 0.003	.018
Reflection	− 0.064	− 0.082	− 0.046	< .001
Reappraisal	− 0.012	− 0.029	0.005	.161
Stressor intensity × rumination	**0.045**	0.034	0.057	** < .001**
Stressor intensity × distraction	− **0.018**	− 0.031	− 0.005	**.008**
Stressor intensity × acceptance	0.007	− 0.005	0.020	.267
Stressor intensity × reflection	− 0.005	− 0.019	0.009	.514
Stressor intensity × reappraisal	0.001	− 0.013	0.014	.890

Random effects were estimated at both levels for elapsed days, lagged NA, and stressor intensity. Bold print indicates significant effects (*p* < .05) relevant for the hypotheses

*NA* negative affect, *CI* confidence interval, *LL* lower limit, *UL* upper limit

In the separate models for each strategy (Table 5), there were significant main effects of all ER strategies in the expected direction: rumination was associated with stronger increases in NA, all other strategies with decreases in NA when stressors had occurred, which we interpret as indicating effectiveness. Furthermore, as to be expected, higher stressor intensity was associated with stronger increases in NA in all five models. Finally, with regard to H2, we found that all strategies with the exception of acceptance interacted with stressor intensity. That is, stressor intensity moderated the relationship between ER strategies and NA for most strategies. More precisely, in the context of more intense stressors (vs. less intense stressors), stronger engagement in reflection, reappraisal, and distraction was associated with a particularly strong decrease in NA, and rumination was associated with a particularly strong increase in NA. For distraction and rumination, these results were in accordance with our hypotheses, but not for reflection, acceptance, and reappraisal. For these three strategies, we had hypothesized to find reduced effectiveness with increasing stressor intensity. Instead, for reflection and reappraisal, we found increased effectiveness with increasing stressor intensity.

In the next step, we modeled all strategies and interactions simultaneously (Table 6). Of the interaction effects found in the separate models reported in Table 5, only the ones for rumination and for distraction remained significant. In both the separate and the combined model, rumination interacted with stressor intensity in a hypothesis-conforming manner: the more intense the stressor, the more rumination was associated with increases in NA. In accordance with our hypotheses, the opposite was true for distraction: the more intense the stressor, the more distraction was associated with decreases in NA. In line with the separate models, the main effect of acceptance and reflection remained significant in the combined model. Thus, the more individuals endorsed acceptance or reflection when a stressor had occurred, the more of a decrease in NA they experienced, regardless of stressor intensity. The main effect of reappraisal did not stay significant, pointing toward reappraisal sharing variance with other predictors in the model — when considering that participants used the other strategies to an average degree, reappraisal had no predictive value above and beyond the other strategies when stressors occurred. Similarly, the interaction effects of reflection and reappraisal did not reach significance, because of shared predictive variance.

To explore whether the relatively strong overlap between reflection and reappraisal (see Table 3) was responsible for the loss of significant interactions in the combined model, we reran the combined analysis including only one or both in the model, but the results stayed similar (with the interaction effects not reaching significance). This implies that the overlap between reflection and reappraisal was not the main reason for the loss of significant interactions, but rather more generally overlap between different predictors in the model.

**Fig. 1:**
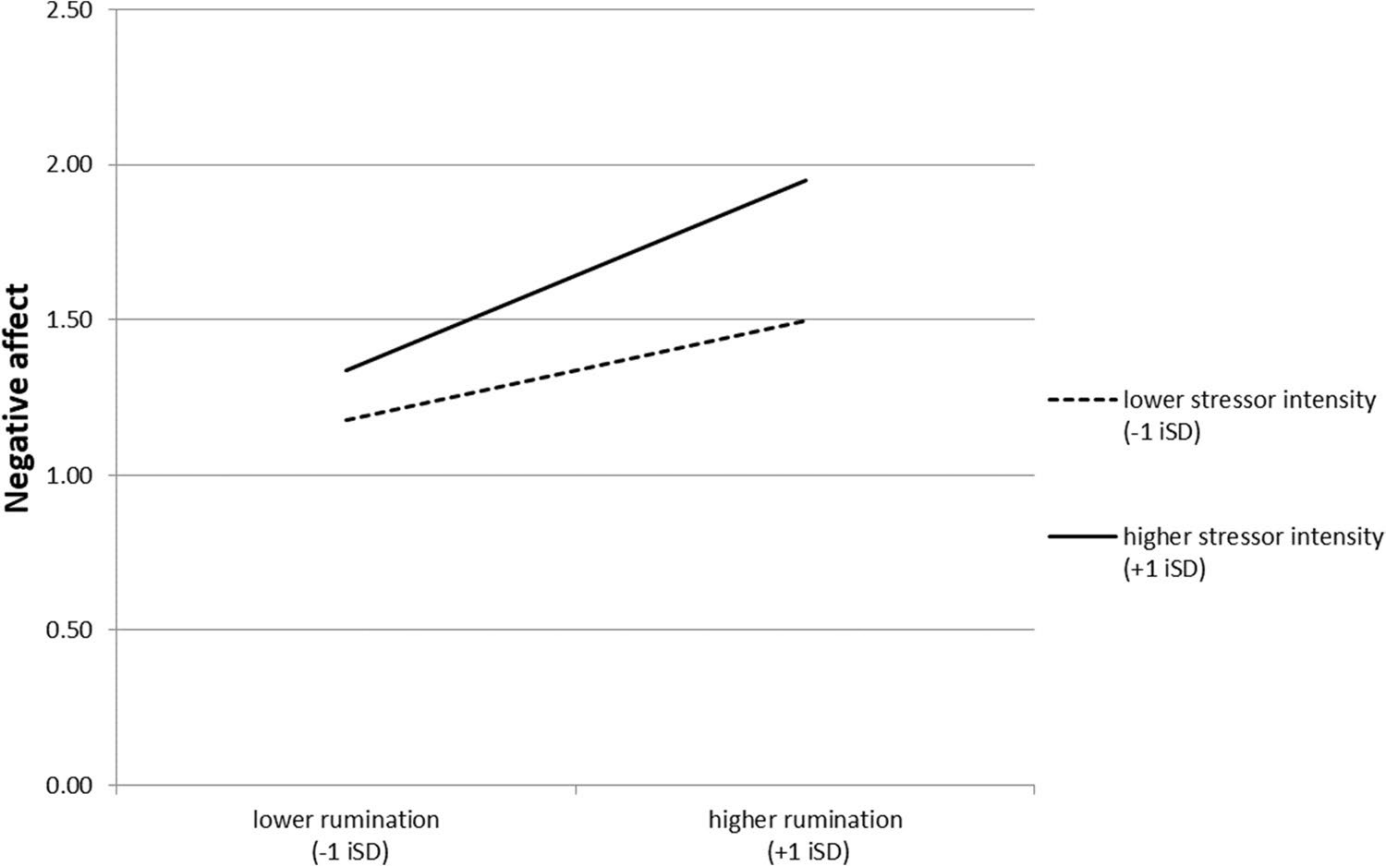
Interaction effect between rumination and stressor intensity in the prediction of NA controlling for lagged NA (change in NA) Note. Illustration of the interaction effect presented in Table 4, controlling for all other effects. NA negative affect, iSD individual standard deviation (at the level of the individual across both waves)

Figure 1 illustrates the interaction effect for rumination (controlling for all other variables in the model). When stressors were of lower intensity, ruminating more was associated with stronger increases in NA than ruminating less. However, when stressors were of higher intensity, this effect was even more pronounced as indicated by the steeper slope.

**Fig. 2:**
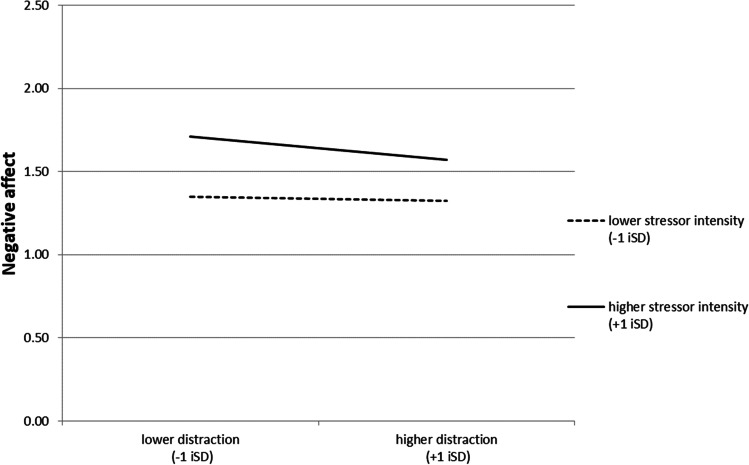
Interaction effect between distraction and stressor intensity in the prediction of NA controlling for lagged NA (change in NA) Note. Illustration of the interaction effect as presented in Table 4, controlling for all other effects. *NA* negative affect, *iSD* individual standard deviation (at the level of the individual across both waves)

Figure 2 illustrates the interaction effect for distraction. When stressors were of lower intensity, it did not matter for NA whether people used distraction more or less. However, when stressors were of higher intensity, endorsing distraction more was associated with stronger decreases in NA than endorsing distraction less.

## Discussion

Building on the framework of ER choice (Sheppes, 2020), we assumed that individuals select and effectively implement strategies depending on the intensity of stressors. In line with our hypothesis, individuals endorsed acceptance, reflection, and reappraisal less when stressors were more intense, potentially because participants lacked the cognitive capacities to use these strategies in stressor-intense contexts (Sheppes, 2020; Veilleux et al., 2021). Against our hypotheses, distraction was not endorsed more when stressors were more intense. Possibly, people may feel like highly intense stressors in daily life (as opposed to the lab) need to be solved rather than distracted from, and/or they are unaware of the short-term effectiveness of distraction. Instead, in line with previous research (e.g., Ortner & Pennekamp, 2020), individuals ruminated more when stressors were more intense, which may be misguided attempts at problem-solving (Nolen-Hoeksema et al., 2008).

With regard to strategy effectiveness, we further hypothesized that all strategies would interact with stressor intensity in their association with change in NA, albeit in different ways: We hypothesized that the effectiveness of distraction and the detrimental effect of rumination would be particularly pronounced when dealing with higher (vs. lower) intensity stressors. Our findings were in line with these predictions, pointing toward an increasingly maladaptive effect of rumination and an adaptive effect of distraction with higher stressor intensity.

Regarding acceptance, reflection, and reappraisal, we expected their effectiveness to be particularly pronounced when dealing with lower (vs. higher) intensity stressors. There was no evidence supporting this. In the separate models, we found evidence for the opposite as reflection and reappraisal followed the same pattern as distraction: that is, these strategies were also more effective when stressor intensity was higher. These results were not in line with the hypotheses made based on the ER choice paradigm. Possibly, participants used these strategies in situations in which they had sufficient cognitive capacities. It may be the case that other factors than stressor intensity (e.g., controllability; see Haines et al., 2016) played a role here as well. When analyzing all strategies simultaneously, interactions with stressor intensity did not remain significant, pointing to some shared effects of the predictors. These differences between the separate and the combined analyses can also help to explain why studies including different strategies may come to different conclusions, as some strategies do not explain variance in NA above and beyond other predictors. In the combined model, acceptance and reflection remained significant as main effects, indicating that these strategies were associated with decreases in NA when stressors had occurred, and suggesting their effectiveness regardless of stressor intensity. For acceptance, similar findings have been found in a laboratory study (Mehta et al., 2017). Since both acceptance and reflection are attentional engagement strategies, they may be easier to use than reappraisal, which requires meaning change (Troy et al., 2018). Reappraisal did not explain variance above and beyond the other strategies. This relatively weaker predictive value of reappraisal may be due to reappraisal being more strongly related to PA than NA in daily life research (see, e.g., Brans et al., 2013; see also supplementary materials).

We considered whether procedural differences between lab and ESM research might explain why the moderating role of intensity on ER effectiveness of reappraisal, reflection, and acceptance was not in line with predictions made based on lab findings. Specifically, in contrast to standardized stressors in the lab, the occurrence and the intensity of the stressors were not manipulated in everyday life in our study. Furthermore, intensity ratings of the stressors in daily life were subjective and continuous. Thereby, we did not compare rather extreme types of stimuli, as is common in the lab (e.g., pictures with high and low intensity). Given that our understanding of the theory behind our hypotheses is that associations between emotional intensity and ER choice and effectiveness are linear (e.g., reappraisal should be more effective when intensity is lower), we think that in principle, one should find evidence for the propositions of the ER choice framework using different research approaches. That is, these procedural differences should not prevent the theoretically plausible effect to occur in daily life. The findings could mean, instead, that the effect only occurs when intensity differences are large.

### Limitations and Future Directions

Our study examined central propositions from the ER choice framework. Other than most previous research on this topic, we used ESM, with the goal to capture stressor intensity variation, ER choice, and effectiveness as ecologically valid as possible and in accordance with prior ESM research. Methodologically, this came with various differences between standard lab designs and our ESM design: As noted above, stressor intensity was not manipulated and differed in various ways from stressor intensity in the lab. Furthermore, everyday stressors also differ with regard to other dimensions than intensity, such as importance (Ortner & Pennekamp, 2020) and controllability (Haines et al., 2016; Wenzel et al., 2020; Wilms et al., 2020). In our study, ER measures also did not depend on stressor occurrence, making it possible that other stressors than those reported were regulated. Moreover, our broader conceptualization of distraction and specific conceptualization of reappraisal (positive) differed from most lab studies. Finally, whereas participants in lab studies make choices on strategies before using them, our time-contingent ESM design included proximal, but retrospective ratings of strategy use regarding multiple strategies, potentially including the use of strategies in combination ("polyregulation"; Ford et al., 2019). As a consequence, a strict comparison of our findings with previous lab findings may be difficult. Additionally, in comparison to experimental manipulations possible in the lab, we cannot make any claims on causality as our analyses were observational and correlational. However, despite the many differences between our study and lab research, some findings could be replicated, which seems promising.

As a solution to these issues, future studies might try to align lab and ESM methodologies to enhance comparability. For example, event-contingent ESM designs could be used, in which participants report on stressors and regulation directly upon occurrence. Also, to learn more about causality using ESM, within-person encouragement designs (Schmiedek & Neubauer, 2020) could be used (e.g., encouraging to use certain strategies in certain situations). However, viewed from a different perspective, comprehensively understanding the phenomenon of ER choice might require a combination of approaches, and diverging findings may yield theoretical advancements. As we outlined, we think that the underlying theoretical assumptions of the ER choice framework should hold even when we use different approaches.

Importantly, we based our study on the assumption that cognitive costs of various strategies differ. However, we did not test this, and systematic evidence regarding the cognitive costs of various ER strategies is not yet available. Furthermore, only a limited number of ER strategies can be assessed in ESM studies, and strategies are usually measured with one item each, which impedes the estimation of within-person reliability (see Brose et al., 2020).

Lastly, we acknowledge that the study of the selection and the effectiveness of ER strategies is theoretically complex. Based on ER choice framework (Sheppes, 2020), we focused on whether the link between ER strategy and NA was directly predicted and/or mitigated by intensity, but future scholars might also explore a mediated path, whereby intensity or other contextual factors predict changes in NA via the use of certain ER strategies. This would require using time-separated variables rather than the approach used here. In our data, other temporal orderings of events are possible: For example, stressor intensity might lead to NA, which then mobilizes other ER efforts to reduce increased NA. Also, ER is not a one-way street; for example, rumination may lead to even more NA, which individuals then have to deal with (Blanke et al., 2021).

## Conclusion

This study sought support for the ER choice framework in daily life. Based on previous research in the laboratory, we investigated whether stressor intensity predicted the selection and moderated the effectiveness of five ER strategies in everyday life. While we did obtain some evidence in favor of stressor intensity playing a role for ER selection and effectiveness, the pattern of results was not as straightforward as could be expected based on laboratory findings. Moving forward, we may thus need to investigate the phenomenon of ER selection and effectiveness from different angles to get a better grasp of the fit between contextual variation and ER strategies.

## Supplementary Information

Supplementary file1 (XLSX 30 kb)

## Footnotes

1 In our study, distraction was not a purely cognitive strategy as we did not specify how people were distracting themselves.

2 We focused on NA in this study, as the measured ER strategies targeted NA. However, results for positive affect (PA) are reported in the supplementary materials (Tables S2b and S3b).

3 A stressor was defined as an unpleasant event of varying intensity. Participants were encouraged to report events of all intensities, including minor events such as missing a bus.

4 On average, the 156 participants completed 69.9 measurement occasions in Wave 1 (*SD* = 6.6; range 30–85) and 68.2 measurement occasions in Wave 2 (*SD* = 10.1; range 10–86). In the following, we included all measurement occasions at which participants indicated that a stressor had occurred. We additionally included occasions at which participants completed the survey only partially (of the total number of included occasions, this pertained to 0.8% in Wave 1 and 1.2% in Wave 2). We kept all participants as part of the sample, including those that provided few data points due to a lack of stressor occurrences. Re-analyzing all analyses with a subset of *N* = 145 participants who reported > 5 stressors across both waves, however, yielded very similar results.

5 To represent the underlying structure of three different NA facets (high arousal NA, high arousal anger domain, low arousal), we let the residuals of the two items of each domain covary when estimating reliability.

6 The wording for reflection was adapted from Brans et al. (2003). However, we were made aware that the phrasing may be confounded with the outcome NA. We repeated our analyses excluding reflection, but the focal results did not change. We therefore decided to not exclude reflection from the analyses.

## Additional information

### Data Availability

Data and materials of Wave 1 are available via the German Institute for Economic Research (DIW Berlin;https://www.diw.de/en/diw_01.c.792406.en/soep-is_innovative_modules.html). Data and materials of Wave 2 will be available in April 2022.

### Code Availability

Code generated within this project will be made available, on the DataverseNL repository upon paper publication (http://companion-is.soep.de/Innovative%20Modules/Adaption%20to%20Major%20Life%20Events.html). All code pertaining to data preparation and analyses is available via the OSF: https://osf.io/6kagp/.
